# From Synaptic Plasticity and Critical Periods to Social Behavior and Stress: Getting to, and Staying in, CA2

**DOI:** 10.1002/hipo.70068

**Published:** 2026-01-10

**Authors:** Serena M. Dudek

**Affiliations:** ^1^ Neurobiology Laboratory National Institute of Environmental Health Sciences, National Institutes of Health Research Triangle Park North Carolina USA

## Abstract

Hippocampal Area CA2, with some exceptions, had long been neglected in in vivo studies, due largely to its small size, and in in vitro studies because of its general similarity to CA3 and CA1. Increasing evidence showing that CA2 was molecularly distinct led to the increased appreciation of CA2 as a separate region, and as such, that it likely had functions that were dissimilar from its neighboring CA subfields that were worth studying. Indeed, it was the molecules that are enriched in CA2 that provided inspiration for many of the functional studies. In this article, I provide a personal account of how I got interested in CA2 and describe how I viewed our discoveries in the context of the others in the field. As it happened, much of my earlier work on synaptic plasticity in hippocampus and visual cortex had everything to do with why I felt compelled to ask the question, “are CA2 synapses resistant to long‐term potentiation?.” In fact, we are a product of our training and our environment when we are considering research directions.

## 1 Introduction

Why would anyone intentionally set out to work on CA2? Mostly, with some exceptions (Bartesaghi and Ravasi 1999), the area had been ignored prior to our work starting in 2005 (Zhao et al. 2007). To be fair, it was not just ignored, not getting a color in illustrations (van Strien et al. 2009), but it was even debated as to whether or not it was a distinct subregion of the hippocampus! My interest in CA2, though, came as a natural extension of my earlier work in hippocampal synaptic plasticity and the study of critical periods in visual cortex. This work led to an initial study in CA2 that eventually took over the focus of my entire lab. That said, I'm quite certain luck also played into it—I doubt I would be working on CA2 had I not been at the NIEHS.

As I'd outlined in the Introduction to the Special Issue on CA2 in *Hippocampus* (Dudek et al. 2023), it was no wonder there was such debate surrounding CA2's identity—none of the molecular markers that marked an area between CA1 and CA3 lined up with the classical definition of CA2 coined by Lorente de Nó (Lorente de Nó 1934; Kohara et al. 2014). Without going into a full review of the literature on CA2—we have written a few (Dudek et al. 2016), I outline here some of what I think were my most significant contributions to the CA2 field. However, I thought it might also be interesting to some researchers to read the history on some of my earlier work, given how it led me to working on this little area of the hippocampus. When considering it does run the length of the hippocampus, CA2 is not *that* small, despite its size relative to CA1 and CA3. So please give it some love as you read this!

An additional note to all my friends and colleagues working in CA2: please forgive me if I have not cited your work (I haven't even cited all of mine). Discussing every paper on CA2 is, at this point, simply not possible given the page limits—and purpose—of this brief review. Instead, my goal with this review is to give the history of how I got interested in CA2 and to summarize my work on the cellular features of CA2.

## 2 chemLTP and LTD

At the University of California Irvine in the 1980s, I had landed a position in Gary Lynch's lab, first as a “199,” where undergraduates could work for course credit (1985–86), and then as a paid research assistant (1986–87). He thought it was hilarious that two Dudeks would come through his lab (F. Edward Dudek was the first. No relation.), so I was in. Mostly, I spent the first year there working with proteins: calpain, brain spectrin/fodrin, calmodulin, and so forth (Seubert et al. 1987), and deciding that I really should apply to graduate school because I was having so much fun doing experiments. A Holy Grail in the lab at the time was to induce Long‐Term Potentiation (LTP) in a hippocampal slice at enough synapses to subsequently allow detection the biochemical changes taking place, and so I was tasked with making that happen during my second year there. I understand that others had tried “rake” electrodes, but we were going straight for the NMDA receptor, as it and post‐synaptic calcium had only recently been discovered to be important for LTP induction in CA1 (Collingridge et al. 1983; Lynch et al. 1983; Alger 2025 and recollections by Collingridge (2025) and Nicoll (2025)). Should be easy to do, right? Wash on the NMDA, get potentiation of the synaptic responses (recorded on our oscilloscope, possibly with a save function and/or camera, but not attached to any computer), freeze for biochemistry. Not exactly though: in maybe a fifth of the slices, this worked as hoped (potentiation), but more than likely I observed what looked like decreases in the responses. With Long‐Term Depression (LTD) not even a consideration at the time, this result could only mean one thing: damage to the slice and/or the beginnings of cell death. So it went: back to the animal facility, cut new slices, repeat with a different concentration of NMDA or time of exposure. I never did get it to work before heading off to Brown University for graduate school to study with Mark Bear, but Olivier Thibault, my successor, was eventually able to induce LTP reliably by adding spermine to the mix (Thibault et al. 1989), though this protocol would never catch on to be put into wide use. Only in later papers, often describing protocols using forskolin, did the terminology “chemLTP” (chemically‐induced LTP) take hold (Otmakhov et al. 2004). Similarly, “chemLTD” was coined in Hey‐Kyoung Lee's studies in Mark Bear's and Rick Huganir's labs (Lee et al. 1998, 2000). It was around then that I realized that yes, in fact, I was likely studying LTD during that time in Gary's lab, before I'd intentionally studied LTD. But this early work in Gary's lab introduced me to slice electrophysiology and protein biochemistry, techniques I still use today.

## 3 mGluRs, LTD, and Ocular Dominance Plasticity

Stay with me here—this does relate to CA2. When I joined Mark Bear's lab at Brown University as his first graduate student (with fantastic undergrads like Dan Feldman and Carlos Aizenman), Mark and I set out to find biochemical differences in visual cortex that could explain the critical periods of postnatal development during which ocular dominance plasticity could occur. Mark's lab in the Center for Neuroscience (I do not think it was a department yet) was largely still under construction when I arrived, so we teamed up with Wayne Bowen, of opiate receptor fame, who had phosphoinositide (PI) turnover assays running in his lab (Bowen et al. 1988). There I was able to do experiments, testing whether glutamate‐stimulated PI turnover was highest early in postnatal development in cortical synaptoneurosomes (it was: Nicoletti et al. 1986; Dudek and Bear 1989; Dudek et al. 1989). Thus formed the basis for my PhD thesis. We had this idea that because NMDA receptors were needed for LTP, mGluRs would be great for mediating LTD (presynaptic activity without the need for postsynaptic depolarization). Trouble was, there were no good protocols for LTD at the time, in 1988, in hippocampus or cortex, even though there was some progress was being made on that front in the cerebellum (Ito and Kano 1982). We were very much inspired by the theoretical work led by Nobel Laureate Leon Cooper that aimed to explain the development of orientation selectivity and ocular dominance plasticity (Bienenstock et al. 1982). A critical part of the Bienenstock, Cooper, and Munro (BCM) theory was that synapses had to weaken, as well as strengthen, in response to presynaptic activity. The model had some similarities with Terry Sejnowski's (Sejnowski 1977a, 1977b), but with some key differences that I won't discuss further here. Mark, with Leon and Ford Ebner, further expanded on the BCM theory to propose a physiological mechanism that would rely on the postsynaptic activity or depolarization (Bear et al. 1987), which was right when I was starting in Mark's lab at Brown. And such began my years‐long quest to get a protocol working to induce LTD. Because I simply could not stomach the unruly squiggly traces that passed as synaptic responses in slices of visual cortex, I'd convinced Mark to let me first work out a method in hippocampus, where we had some confidence that field potential recordings reflected excitatory synaptic responses. About the same time that I was going about trying a number of different protocols using low frequencies of stimulation, Patrick Stanton, with Terry Sejnowski, had published a method of inducing LTD using synaptic stimulation that was anti‐correlated with bursts of stimulation (Stanton and Sejnowski 1989). Of course I was excited to try out this protocol. However, we and others tried it with no success (likely Pat was looking at de‐potentiation) (Paulsen et al. 1993). It made some sense to model the BCM theory experimentally by using low frequencies of presynaptic stimulation instead of small amounts of postsynaptic depolarization (though I was wanting to eventually try intracellular recordings to directly depolarize neurons). Additionally, there were hints from Kristen Harris's work in Tim Tyler's lab that a developmentally dependent synaptic depression was actually possible (Harris and Teyler 1984). Mostly though, it just took lengthening the period of stimulation to 900 pulses (15 min at 1 Hz or 30 min at 0.5 Hz) until the depression “stuck” (i.e., the synaptic responses did not return to the baseline amplitudes). I don't recall when exactly it finally clicked, but I'd first presented it at an SfN meeting, I think in the fall of 1991. I also recall excitedly driving down to San Diego from my parents' house in Anaheim while I was on Christmas break to share some of my first experiments with Terry Sejnowski (with Mark's permission). He (Terry) seemed to appreciate the work, but the same could not be said about everyone at that SfN meeting! Best I could tell though, for the most part, most of us studying LTD back then were on friendly terms, and there were several other groups interested in LTD in the visual cortex (Artola et al. 1990; Fregnac et al. 1994). We published our first paper on NMDA receptor‐dependent LTD in the hippocampus in May of 1992, right as I was defending my Ph.D. dissertation (Dudek and Bear 1992). If you look closely at the manuscript, you may notice that it was communicated directly to PNAS by Leon Cooper. I cannot say for sure why Mark decided to go that route (admittedly, it was only lightly reviewed, and required only minor revisions, so it was in contrast to my experiences in publishing on my own—see below). I suspect, though, that it was because Rob Malenka was hot on our heels, publishing their work on the role of phosphatases in LTD in November of 1992 after hearing of our results from Mark earlier in the year (or so I'd heard) (Mulkey and Malenka 1992).

An additional roadblock to testing our idea of a role for mGluRs in LTD was that although the antagonists of mGluRs were just being discovered (Eaton et al. 1993; Ikeda 1993) (Collingridge 2025), MCPG required very high concentrations and AP3 was thought to be non‐selective. Thus, demonstrating that my LTD protocol (900 pulses delivered at 1 Hz) required mGluRs did not happen during my time in the Bear lab (that would be Kim Huber's achievement using a slightly different stimulation protocol (Huber et al. 2001)). However, unexpectedly, the NMDA receptor antagonist APV blocked it (Dudek and Bear 1992). While somewhat confused and profoundly disappointed that it was not mGluR‐dependent, we were quite happy to see that something, *anything*, like a drug could prevent it, and that it was not an artifact of the electrical stimulation like the isolation unit battery running out. C'est la vie. Happily though LTD was more robust early in postnatal development as we'd predicted (Dudek and Bear 1993), and around the same time, visualized patch clamping in slices from young animals became the norm. As subsequent work from Mark's lab has shown, the idea of LTD (mGluR or NMDA‐receptor dependent) playing a role in ocular dominance plasticity during critical periods of development is still very much alive and well (Crozier et al. 2007).

**FIGURE 1:**
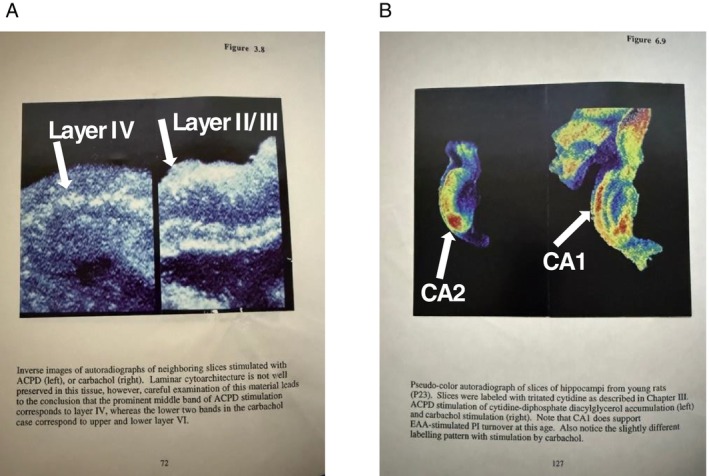
Similarity between layer IV of kitten primary visual cortex and area CA2 of rat hippocampus. Unpublished images from my PhD dissertation showing distribution of agonist‐stimulated phosphoinositide turnover (laboratory of Mark Bear, Brown University, 1992). See Hwang et al. (1990), for methods. (A) Slices of kitten visual cortex (postnatal day 36) were incubated with either the Group I and II metabotropic glutamate receptor agonist ACPD (left) or the cholinergic receptor agonist carbachol (right). ACPD‐stimulated accumulation of label was primarily in layer IV, whereas carbachol‐stimulated label was primarily in layers II/II and V and VI. (B) Slices of hippocampus from a rat, postnatal day 23, stimulated in the same way with ACPD (left) or carbachol (right). Consistent with Hwang et al. (1990), ACPD‐stimulated accumulation of label was concentrated in area CA2, which was different than that stimulated by carbachol (CA1 and CA3 mostly). Note that the ^3^H accumulation does not distinguish between presynaptic terminals from external sources (input from the lateral geniculate nucleus in the case of layer IV, for example) and postsynaptic dendrites. Labels were added in this manuscript.

Funny though—unpublished results of other experiments that were part of my graduate work had hinted at CA2 being different from CA1 and CA3—results that we *all* summarily ignored (we meaning Mark, me, and anyone studying PI turnover). I had been replicating work by Sol Snyder's lab showing the spatial profile of agonist‐induced PI turnover in visual cortex. This technique involved incubating brain slices in tritiated cytidine, which upon stimulation with different agonists, would be incorporated into the membrane‐bound diacyl glycerol (Hwang et al. 1990). Upon drying the slices and exposing them to x‐ray film, one could determine where the PI turnover was occurring. Although I was focusing on visual cortex, I also included hippocampus as a way to tie it into my LTD work (Figure 1). Unfortunately, as sometimes happens, the experiments stopped working for mysterious reasons, and I didn't have time to troubleshoot the problem. Only upon cleaning my office many, many years later, and finding the figures from my dissertation, did I see clear as day: the Group I and II mGluR agonist 1‐amino‐1,3‐dicarboxycyclopentane (ACPD) had stimulated a pattern of radioactivity deposition that was distinct from that stimulated by carbachol, a muscarinic acetylcholine receptor agonist (Hwang et al. 1990). Right. There. In. CA2.

## 4 Regulating Critical Periods and TREKs

The advice I now often give to trainees is this, “Always attend departmental seminars. You never know where your great ideas will come from.” In my case, it was one such seminar that led me to CA2 for real. I began my lab at NIEHS in 2001 with the intention of finding molecules that increase over development but would *prevent* plasticity, or close, the so‐called critical period. I was not alone in the field, and Carla Shatz's and Mriganka Sur's labs were completing microarray‐type studies to get at that question (Huh et al. 2000; Lyckman et al. 2008). Additionally, perineuronal nets (PNNs), a specialized extracellular matrix, were by then already being appreciated as a “closer” of critical periods (Pizzorusso et al. 2002). I was on the lookout for similar such molecules that were highest in adult layer IV, as it had been shown that ocular dominance plasticity ends earliest and most completely there (Mower et al. 1985), and I'd shown in my postdoctoral work that LTD in layer IV also disappears with age (Dudek and Friedlander 1996), which was not the case for other, more superficial cortical layers (Kirkwood et al. 1993). The “a‐hah moment,” though, came when my colleague here at NIEHS, David Armstrong, had invited Doug Bayliss to present a seminar on the two‐pore domain potassium channels (TREKs and the like). The talk was on respiratory neurons, but I could not help noticing on one of his slides that one of the TREKs was highest in layer IV, and so I was taking notes! But then he showed a close‐up of the hippocampus. “Huh. That's strange.”, I thought. TREK‐1 expression was super high in what looked like CA2 (Talley et al. 2001). I, like many in the field, did not know there was such a thing as a distinct CA2, but it sure seemed that if TREKs were preventing plasticity in layer IV, they might similarly be preventing it in CA2. Later, I'd also found a paper showing that TREKs increase with postnatal age (Kanjhan et al. 2004), which is what we might expect if they are inhibiting synaptic plasticity. So the simple prediction I made that day was that synapses in CA2 would not have LTP or LTD like synapses in CA1 and CA3. The idea was easily testable, and there was also the added benefit that pyramidal neurons in CA2, unlike neurons in layer IV, had input from the same presynaptic neurons (CA3), as those in CA1, and were a heck of a lot easier to patch! Indeed, no matter what we did, like adding GABA receptor blockers, increasing the frequency of stimulation, or raising calcium (though more on that later), we could not induce LTP (Zhao et al. 2007), though LTP was easily induced in CA1. TREKs did not appear to be involved, however. We never returned to visual cortex.

### 4.1 Adventures in Publishing (1)

To me, this was the coolest finding since LTD: CA2 synapses in *stratum radiatum* did not show LTP like those in CA1 in response to typical high frequency stimulation or pairing protocols. We found a lot about what was *not* likely to explain this lack of LTP for that first study, but we were stuck with a cool observation without finding the “mechanism.” There were several, possibly redundant, mechanisms, but finding *any* mechanism could take years. One editorial rejection came back saying this was a “negative finding”! One high impact journal did review our paper, but the reviewers were having none of it: some critiques were easily addressable (using perforated patch, we ruled out that dialyzing the cells was the reason for the lack of LTP—though why they thought this might happen in the larger cells of CA2 but not the small ones in CA1 I do not know). Another critique was “how do you know you're in CA2”? Maybe it is a bit of a circular argument, but the obvious answer was that we *can* get LTP in CA1 and CA3. So, if we strayed out of CA2 and into CA1 (or CA3), our average effect size would be considerably decreased (sigh). At the time we did not have great antibodies marking CA2, and my postdoc at the time (Meilan Zhao) was not having any luck recovering filled cells to confirm their location (although Audrey Mercer, working with Alex Thompson, was doing just that with sharp electrodes and using an antibody to alpha actinin 2 (Mercer et al. 2007)). It was fortunate, though, that around then I had visited Karl Obrietan, and he showed me some great staining for STEP phosphatase, which is particularly enriched in CA2 (Boulanger et al. 1995), and some staining showing (a lack of) phosphorylation of ERK in CA2 after seizures. I thought that might provide a bit more physiological relevance, and so we added that to the manuscript. In the end, the seizure data got moved to supplemental data because we were not really making any conclusions about CA2 and seizures, but we think it gave the reviewers and editors at *J. Neuroscience* some confidence that the lack of LTP in CA2 was likely to be real (Zhao et al. 2007).

## 5 Regulators of Plasticity in CA2

Looking back, I know it was naïve of me to think it was just that one ion channel like TREK that was preventing LTP in CA2. At some point, I'd become aware of earlier work by Ed Lein and Fred Gage and others showing that *many* genes were highly expressed in CA2 (Zhao et al. 2001; Lein et al. 2004). But I can say TREK1 got us there. Our first real breakthrough on mechanism, though, would have to wait for me to be contacted by John Hepler, who was studying the regulator of G‐protein signaling (RGS)‐14 (RGS14). They had an RGS14 knockout mouse, but had seen no effect of knockout on LTP in CA1. Although the staining was most obviously in CA2, they, like others even today, had not thought to look in CA2. One of my friends who saw his seminar mentioned to him to talk with me about testing LTP in CA2. The effect of RGS14 knockout was an amazing “restoration” or “enabling” of LTP in CA2 (Lee et al. 2010). It was almost too easy. This was great. Incidentally, I first presented this work at a meeting of the 2010 Molecular and Cellular Cognition Society, right after Nobel prize winner Eric Kandel. Nothing more intimidating than speaking to a packed room as a tenure track investigator! RGS14 is now one of our favorite stains for CA2 pyramidal neurons, and it made another appearance in the lab many years later as being critical for mGluR‐LTD in CA2 (Samadi et al. 2023). So knockout of RGS14 really appears to shift the “plasticity profile” in CA2 from one where synaptic depression is prominent, with no LTP to speak of, to one with no depression and typical LTP.

It seemed at that time worth investigating other potential regulators of plasticity in CA2—did they do anything interesting? Several CA2‐enriched proteins pointed at calcium buffering being different (Necab2, PCP4, etc.), and so my postdoc Steve Simons was wanting to know if calcium signaling in dendritic spines was “normal” (i.e., similar to that in CA1) and was wanting some experience imaging. We therefore teamed up with Ryohei Yasuda when he was at Duke University nearby. Steve and Ryohei found that CA2 spines and dendrites had only about ¼ of the peak calcium levels as what is seen in CA1, but that greater calcium extrusion, not buffering, was likely the driver of the difference (Simons et al. 2009). Steve, with a biomedical engineering background, suggested we try 10 mM calcium (vs our normal 2.5 mM) in our artificial cerebral‐spinal fluid (ACSF) to overcome the robust buffering and extrusion in CA2 pyramidal cells. This to me was insane for a few reasons: (1) we had already tried 4 mM as part of our first paper, without getting LTP, and (2) if RGS14 is still around, why would increasing calcium be able enable LTP? Of course it worked and it enabled LTP in CA2 (Simons et al. 2009). This was not the first time I would be proven wrong when it came to CA2—the pattern would repeat itself with PNNs as well: enzymatic disruption of PNNs “enabled” LTP in CA2 (Carstens et al. 2016). I found myself thinking of the situation with ocular dominance plasticity—there were several ways to “re‐enable” plasticity after the critical period had ended. Though how could any one mechanism be essential? We can only presume that this finely tuned plasticity‐regulating machine (molecular complex?) that prevents LTP in CA2 falls apart with removal of any one component. I still do not know what to make of this. Adding to the puzzle is that while all our work had been performed in the stratum radiatum, Vivien Chevaleyre in Steven Siegelbaum's lab had found that he could get LTP in the distal dendrites (Chevaleyre and Siegelbaum 2010) (*stratum lacunosum moleculare*; SLM), with no obvious *lack* of RGS14 in the SLM, for example. Shannon Farris, a former postdoc in my lab, now with her own lab, has shown that mitochondrial calcium uniporters are enriched in the CA2 SLM and may help explain some of these differences (Pannoni et al. 2023).

Basically while we were going down our list of potential plasticity regulators in CA2, we found that to my surprise, caffeine and other A1 adenosine receptor antagonists, as well as vasopressin and oxytocin, could *induce* potentiation in CA2 neurons (Simons et al. 2011; Farris et al. 2019). Oddly, in the case of A1 adenosine receptors, we see no evidence of *Adora1* gene expression being enriched in CA2 (Farris et al. 2019), despite nice staining there being reported (Ochiishi et al. 1999). We tried unsuccessfully to get the antibody, and none of the commercial antibodies showed the pattern indicating A1Rs being highest in CA2. Thus, the antibodies to A1Rs join the infamous group of reagents that stain CA2 with no evidence of the gene being highly expressed there (alpha actinin 2 and delta opioid receptors being others (Wyszynski et al. 1998; Burstein et al. 2013)). However, we did note that expression of several adenylyl cyclases was greatest in CA2, as well as “de‐enrichment” of several phosphodiesterases (Farris et al. 2017; Caruana and Dudek 2020), which gave us confidence in the findings. In a second series of experiments, we found that both oxytocin and vasopressin were able to induce a sort of potentiation as well (Pagani et al. 2015). Thus began the idea that “social neuropeptides” could regulate synaptic plasticity in CA2 (more on this later). This study was the result of Scott Young's gentle badgering *for years* for us to put vasopressin on our slices: they had found that the gene encoding the vasopressin 1b receptor (*Avpr1b*) was selectively expressed in CA2 and the *Avpr1b* knockout mice had reduced social aggression (Wersinger et al. 2002). Despite the interesting backstory, peptides in general were unappealing to me to study because they were known to be slow acting and were thought to be “sticky” in thick tissue slices, or otherwise simply by virtue of them acting through G‐protein coupled receptors (GPCRs), and so it was quite a bit lower than the others on our to‐do list. After completing our other studies on RGS14 and caffeine, I finally relented, and we were rewarded with a synaptic potentiation in CA2 with vasopressin and with oxytocin, another Gq type of GPCR. Oxytocin receptors are also highest in CA2 but had a bit more expression in the distal CA3, unlike *Avpr1b*, which has expression gloriously selective for CA2 such that one of the better cre‐mouse lines uses *Avpr1b*. Interestingly, the peptide‐induced potentiation and the caffeine‐induced potentiation had several key differences: whereas caffeine‐induced potentiation was rapidly induced, sensitive to intracellular dialysis, and was cAMP and PKA dependent, the potentiation induced by vasopressin and oxytocin developed slowly, required synaptic stimulation, NMDA receptor activation and post‐synaptic calcium, very much resembling canonical LTP (Nicoll 2017). Several other peptides like Substance P induced potentiation in a similar way (Dasgupta et al. 2017).

As summarized in one of our review articles (Carstens and Dudek 2019) and work from Elizabeth Gould's lab (Diethorn and Gould 2023), most of these potential negative regulators of synaptic plasticity increase with postnatal age in rodents in that they are barely if at all detected until the second postnatal week (Laham et al. 2021; Diethorn and Gould 2023). Most of our initial studies were performed around postnatal day 14 (P14), which was quite a bit earlier than the ending of other critical periods like visual cortex, which ends around postnatal day 30 in mice (Gordon and Stryker 1996). So one question that remained was whether one *could* induce LTP prior to the developmental expression of most of our “plasticity regulators” RGS14 and PNNs. In fact, we found that we could induce something more like “typical” LTP in the P8‐11 range (Carstens, Lustberg, et al. 2021). Although we are not pursuing further this idea of critical periods in hippocampal function, we did find that the period during which plasticity could be induced in CA2 is prematurely ended in a mouse model of Rett Syndrome (*Mecp2* null), likely due to the early development of PNNs there (Carstens, Lustberg, et al. 2021). In addition, similarly premature development or enhanced expression of PNNs had been found in other mouse models of autism including the BTBR mouse line (Cope et al. 2022) and Engrailed2 knockout mice (Mattioni et al. 2024), found by Elizabeth Gould's and Giovanni Provenzano's labs respectively. Together, these findings suggest that, in rodents anyway, a suite of ‘plasticity regulators’ all increase in tandem in CA2 during postnatal development to give CA2 its distinct molecular profile. That the plasticity profile in CA2 (i.e., the ability to induce LTP) is *mostly* similar to that in CA1 and CA3 during the first postnatal week hints at something like a period during which circuitry is established in a way that is typical of the rest of the hippocampus, prior to taking on distinct synaptic properties starting in the second postnatal week.

As part of our study looking at the *Mecp2* null mice, we figured that the enhanced PNN staining was due to the hyperexcitability of the hippocampus and seizure activity as previously described (Calfa et al. 2015). Apparently, this was not the case. Two pieces of evidence suggested that the opposite is true: that CA2 could be hypoactive in these mouse models of developmental disorders. First, using designer receptors exclusively activated by designer drugs (DREADDs) to chronically inhibit or excite CA2 neuron function, we found an *inverse* relationship between activity and PNN staining—that is—increased activity led to decreased PNNs, and decreased activity led to increased PNNs (Carstens, Lustberg, et al. 2021). Second, using a mouse that displayed a developmental onset of seizure activity, we found a *decrease* in PNN staining. Together, these findings suggested that CA2 is hypoactive in some mouse models of human developmental disorders.

## 6 Sloppy, Unstable Place Fields, and Social Behavior

Honestly, around 2011, we were beginning to think that no one else was interested in CA2 besides us, even though I would talk about our work on CA2 to whoever would listen; few had any interest in recording from there in vivo given its small size. Therefore, we knew that at some point, we were going to need to drop an electrode into a live animal to find out what these neurons do. We had suspected, based on Scott Young's work showing that vasopressin receptors were highly expressed in CA2 (Young et al. 2006), that the neurons might respond to social stimulation. Mostly though, I just wanted to see how their firing might differ from those in CA1 and CA3. At the same time, optogenetics and DREADDs were becoming more popular, and so I knew we had to similarly place a priority on getting genetic access to CA2 using cre‐recombinase. Fortunately, two events that were critical to many of my lab's subsequent successes occurred. First, Geogia Alexander applied for a job I'd posted. She was the first author on Brian Roth's first paper on expressing DREADDs in mice (Alexander et al. 2009), and so she could have easily found a faculty position to pursue her own interest, epilepsy. For several reasons, though, she was needing to stay in the area, and as I had just received tenure, I was able to hire her. Second, my colleague here at NIEHS, Patricia Jensen, had already made the construct to generate a transgenic *Amigo2*‐(CA2)‐cre line and they collaborated with us to get those going. As we felt that any opto‐ or chemo‐genetic manipulation of CA2 neurons would require some knowledge of CA2 neuronal activity in a behaving animal, Georgia spent most of her time recording (in rats), and only secondarily breeding and characterizing the mice, which were new for us. Splitting her time like that likely contributed to us getting scooped on both the silencing (Hitti and Siegelbaum 2014) and the place fields (Mankin et al. 2015)! Not complaining here: we did publish first showing place field remapping in response to social stimulus and the use of DREADDs in CA2 was new (Alexander et al. 2016; Alexander et al. 2018). I did like to think I had some small part in inspiring Steven Siegelbaum's lab to look at social behavior, though; I'd presented our vasopressin work as a poster at a Society for Neuroscience (SfN) meeting and did talk to his graduate student Fred Hitti at my poster. Of course, ideas are cheap, and Steven beat both mine and Scott Young's groups on the social recognition memory work. Subsequent work by the Siegelbaum lab and others have really advanced the circuit‐level role of CA2 (Leroy et al. 2018; Donegan et al. 2020), and they are (now) too numerous to mention here.

### 6.1 Adventures in Publishing (2)

Although we had some great examples of cells that appeared to fire in response to social stimulation using tetrode recordings in CA2, they were uncommon. More often though, cells showed firing that resembled place fields with a few important distinctions. First, the cells had a higher average firing rate, likely due to the place fields being larger than those in CA1 and CA3. Strikingly though, they were fundamentally unstable, leading the Leutgeb lab to interpret this property as a mechanism for encoding time (Mankin et al. 2015). We had read their SfN abstract and tried hard to recruit graduate student Emily Mankin to come work with us. Alas, she went on to non‐CA2 work, but we did learn that they had submitted a manuscript that would soon be published. Although we'd submitted our paper on the CA2 place fields remapping in response to social and novel stimuli prior to their paper being published, the editors no longer thought our results met the threshold for novelty (really!?). As it turned out, Laura Colgin was finding similar results in their recordings of CA2 neurons, and so we teamed up to put together a stronger manuscript (Alexander et al. 2016). These results showed again that something interesting is going on in CA2 and that social cognition may be one of CA2's critical functions. I still think CA2 is more interesting than “just” social cognition; we found place field remapping in response to novelty as well, suggesting that processing information on salience might also be part of CA2 repertoire.

### 6.2 Adventures in Publishing (3)

Around the same time, we were gearing up to put DREADDs in CA2. We found that activation of excitatory DREADDs, when expressed in dorsal CA2, increased gamma oscillations and decreased sharp wave ripples in the hippocampus and in the prefrontal cortex, as well as increasing coherence between them (Alexander et al. 2018). We also found that inhibition of CA2 pyramidal neurons with inhibitory DREADDs impaired social recognition memory—*only in male, but not in female* mice. In an all too familiar situation, we had gone back and forth with reviewers—one thinking the sex difference in the behavior was super interesting, deserving of a main figure, and the other one later noting that we were making too much of this sex difference in behavior without an accompanying difference in the electrophysiology (although we had recorded from both male and female mice, we had not powered the experiments to detect sex differences). The paper was ultimately rejected. As we were not prepared to pursue the mechanisms underlying the sex differences in behavior—it could be anywhere downstream of CA2 after all—we took all reference to the behavior out in our next version that was eventually published (Alexander et al. 2018). If you are interested though, you can find our behavior from males and females in the original manuscript posted on BioRxiv (Alexander et al. 2017). Sex differences would show up again in our study on contextual fear memory, where it was only the female mice that were impacted by manipulating CA2 activity (Alexander et al. 2019).

## 7 On CA2's Molecular Phenotype and the Stress Hormone Receptor Controlling It

As part of our earlier manuscript looking at place fields, Shannon Farris in the lab looked at immediate early gene (IEG) expression in CA2 in response to social experience. I recall presenting preliminary results at the Spring Hippocampal Research meeting and saying that “CA2 neurons had no IEG induction”. In fact, upon careful examination, Shannon found that yes, one could find transcriptional foci labeled for *Arc*, though they were much smaller than elsewhere and easily lost in the background (Alexander et al. 2016). Although not mentioned in the manuscript, this contrasted with *Fos*, which showed no induction at all, making it wholly unsuitable for many of the “engram”‐type technologies now used to query memory circuits (Franceschini et al. 2025). As an aside, we noted during the experiments that the animals spent more time exploring a social stimulus and *not* exploring the arena, which is one explanation for the delay in *Arc* induction we observed in the social experience condition. We still think this reduced/lack of IEG expression in CA2 could be explained by the expression of the same molecules that may be preventing LTP in CA2 are preventing IEG expression (for example—STEP is an ERK phosphatase highly expressed in CA2, and ERK activity is required for IEG transcription and LTP (Boulanger et al. 1995; Pelkey et al. 2002; Paul et al. 2003)). However, another possibility is that LTD in CA2 is prominent, and IEGs are just not induced, despite the cells firing. In an effort to get a full view of CA2's molecular characteristics using modern sequencing methods, though, Shannon took on the project. As she was also interested in dendritic mRNA, we opted for laser capture microscopy and RNA‐seq. Arrgh! Scooped again! Janelia's Hipposeq, led by Mark Cembrowski in Nelson Spruston's lab, published the RNA‐seq database from all the hippocampal regions, including CA2 (Cembrowski et al. 2016). Nevertheless, Shannon did find an interesting enrichment of mitochondrial calcium uniporters in CA2 dendrites (Farris et al. 2019), and she has now pursued that further in her own lab (Pannoni et al. 2023, 2025). I'm thrilled that our bioinformaticist, James Ward, also developed a web‐based application called SpliceJam, where this data can be queried for any gene's expression profile in the hippocampus—cell bodies or dendrites—and splicing (Farris et al. 2019). We still use this data set for our “ground‐truth” when comparing our results using other spatial transcriptomics technologies.

Fast‐forward a few years, and we found ourselves dragged into the world of steroid hormones. Of course even before we had our RNA‐seq data in hand, I had been compiling my list of CA2‐enriched molecules that I was collecting from the literature I'd encountered. One of those was the mineralocorticoid receptor (MR) that was visible in some of the early in situ hybridization studies from Huda Akil's lab and immunostaining from Robert Spencer's lab (Herman et al. 1989; Kwak et al. 1993; Kalman and Spencer 2002). We knew we would need a knockout for our electrophysiology, and it was fortunate that our post‐bac trainee, Danny Lustberg, “acquired” some of the conditional MR knockout mice from another investigator at NIEHS (technically this was a collaboration at the time, and the mouse was given to him by a post‐doc in the other lab). What Danny found was striking: a complete loss of all CA2 markers in mice lacking *Nr3c2*, the gene encoding MRs (McCann et al. 2021)! Our recent work using spatial transcriptomics has expanded on this finding to confirm our impression that the neurons in the CA2 area in the conditional MR knockouts take on many of the molecular characteristics of CA1 (Harris et al. 2025).

### 7.1 Adventures in Publishing (4)

To me, this was THE most astounding discovery, well—since we found CA2's lack of LTP! Yet an early version of the manuscript, formatted as a brief communication, was not even reviewed. Why would the editors of the high‐profile journals not want this? Upon a few discussions with editors, I learned that they'd want either the dreaded “mechanism” (we still do not know this!) or behavioral studies. So we performed the latter and found that CA2targeted knockout of *Nr3c2* was sufficient to mimic many of the behavioral results first reported in previous work using *CaMKII*‐cre mice, including social recognition memory and reactivity to novelty (Berger et al. 2006; Ter Horst et al. 2014). Alas, the manuscript was not even reviewed at said journal.

## 8 Recent and Ongoing Studies—PNNs, AAVs, Stress, and Autism

Because enzymatic degradation of PNNs does not distinguish between the CA2 pyramidal cells and the inhibitory interneurons, even when we restricted expression of chondroitinase to cre‐expressing CA2 neurons (Carstens, Gloss, et al. 2021), we sought to develop mice lacking a primary protein component of PNNs, aggrecan, in either CA2 pyramidal neurons or parvalbumin positive interneurons to probe the respective behavioral effects (Alexander et al. 2025). To do this we made a floxed *Acan* mouse line and crossed them to either our *Amigo2* (CA2)‐cre or *Parv*‐cre lines. We found that the CA2‐targeted *Acan* knockout, but not the brain‐wide parvalbumin cell knockout, resulted in social memory deficits (Alexander et al. 2025). What we (“we” meaning Georgia again) also found was a signature of social exploration in control mice but not in our CA2‐targeted *Acan* knockout mice: a shift up in the theta frequency when an animal explored the novel social stimulus, which appeared to shift back down upon exploration of the now familiar animal. We think that because the presumed input from the Supramammillary nucleus (SuM, labeled with VGluT2) was not entirely normal in the knockout, the signal may be coming from the SuM. Experiments exploring the mechanisms underlying this shift in theta are underway.

Really, one of the things we wanted to test with these *Acan* conditional knockout mice was to determine whether PNNs were responsible for CA2's peculiar affinity for Adeno‐associated viruses (AAVs). We and others had noticed that even the AAV serotypes crossing into the brain from the bloodstream like AAV‐PHP and ‐PHP.eb seemed to target CA2 (Chan et al. 2017; Simonnet et al. 2023). Contrary to our hypothesis, this affinity was *not* due to the PNNs, but was due to the high expression of an AAV receptor, as well as a number of other likely co‐receptors, in CA2 (Alexander et al. 2024). By the way, if you are looking to *avoid* CA2 expression of AAV carried cargo, AAV6 is the one to go with; it appears to stay true to its injection site.

Finally, I find that we are focusing more and more on the MR and its function in CA2 given its apparent role as a “master regulator” of 'CA2 gene' expression. We did not see obvious differences in neuron excitability or synaptic function in the knockouts other than the observed “enabling” of LTP, which was not surprising with the loss of all of our demonstrated and presumed negative regulators of LTP and the CA1‐like gene expression pattern. Do MRs regulate CA2 gene expression in CA2 in response to stress—even though their high affinity for corticosterone has been thought to render them active in the non‐stressed case? Do they have some special role in CA2 compared with CA1 and CA3? We found that MRs and RGS14 are down‐regulated with prolonged exposure to corticosterone (McCann et al. 2021), so is that their primary role—to respond to chronic stress? And how do the human variants of *NR3C2* found in autism impact CA2 function (Ruzzo et al. 2019; Cukier et al. 2020)? Stay tuned.

Reflecting back on my now 25 years of working on CA2, I'm reminded of not only just how much we have learned about the molecular and cellular regulation of synaptic plasticity in CA2 in those 25 years (Carstens and Dudek 2019), but also of just how much we still have to learn at that level. For example, one nagging question is, how can each of the negative regulators of LTP (RGS14, robust calcium handling, perineuronal nets) be truly critical? As in, why would knockout of RGS14 enable LTP when the PNNs are still present? Do the positive regulators (oxytocin and vasopressin receptors, together with other GPCRs) disable the negative regulators? However, it is also clear that research on CA2 neurons at the circuit level is decades behind that of other hippocampal regions. Enrichment of the social neuropeptide receptors in CA2 was a clue leading us to look at CA2 in the context of social behavior, but can we gain similar insights from other enriched molecules like the mineralocorticoid receptor, which might suggest a role for CA2 in stress hormone processing? Does CA2 function depend on synaptic plasticity (LTP OR LTD) at all? Thus, I consider myself still in an “exploration mode”: following up on the many clues that the distribution of molecules in CA2 is handing us.

## Funding

This work was supported by National Institute of Environmental Health Sciences (NIEHS Z01‐ES100221).

## Conflicts of Interest

The author declares no conflicts of interest.

## Data Availability

Data sharing not applicable to this article as no datasets were generated or analyzed during the current study.

## References

[hipo70068-bib-0002] Alexander, G. M. , L. Y. Brown , S. Farris , et al. 2017. “CA2 Neuronal Activity Controls Hippocampal Oscillations and Social Behavior.” bioRxiv. 10.7554/eLife.38052 PMC6251629 30387713

[hipo70068-bib-0001] Alexander, G. M. , L. Y. Brown , S. Farris , et al. 2018. “CA2 Neuronal Activity Controls Hippocampal Low Gamma and Ripple Oscillations.” eLife 7: e38052. 30387713 10.7554/eLife.38052 PMC6251629

[hipo70068-bib-0003] Alexander, G. M. , S. Farris , J. R. Pirone , C. Zheng , L. L. Colgin , and S. M. Dudek . 2016. “Social and Novel Contexts Modify Hippocampal CA2 Representations of Space.” Nature Communications 7: 10300. 10.1038/ncomms10300 PMC4737730 26806606

[hipo70068-bib-0004] Alexander, G. M. , B. He , A. Leikvoll , et al. 2024. “Hippocampal CA2 Neurons Disproportionately Express AAV‐Delivered Genetic Cargo.” bioRxiv.

[hipo70068-bib-0005] Alexander, G. M. , V. D. Nikolova , T. M. Stober , A. Gruzdev , S. S. Moy , and S. M. Dudek . 2025. “Perineuronal Nets on CA2 Pyramidal Cells and Parvalbumin‐Expressing Cells Differentially Regulate Hippocampal‐Dependent Memory.” Journal of Neuroscience 45, no. 6: e1626242024. 39740999 10.1523/JNEUROSCI.1626-24.2024 PMC11800750

[hipo70068-bib-0006] Alexander, G. M. , N. V. Riddick , K. E. McCann , D. Lustberg , S. S. Moy , and S. M. Dudek . 2019. “Modulation of CA2 Neuronal Activity Increases Behavioral Responses to Fear Conditioning in Female Mice.” Neurobiology of Learning and Memory 163: 107044. 31319167 10.1016/j.nlm.2019.107044 PMC6689262

[hipo70068-bib-0007] Alexander, G. M. , S. C. Rogan , A. I. Abbas , et al. 2009. “Remote Control of Neuronal Activity in Transgenic Mice Expressing Evolved G Protein‐Coupled Receptors.” Neuron 63, no. 1: 27–39. 19607790 10.1016/j.neuron.2009.06.014 PMC2751885

[hipo70068-bib-0008] Alger, B. E. 2025. “Notes on the History of In Vitro Hippocampal Electrophysiology and LTP: Personal Reflections.” Hippocampus 35, no. 6: e70047. 41165256 10.1002/hipo.70047 PMC12574198

[hipo70068-bib-0009] Artola, A. , S. Brocher , and W. Singer . 1990. “Different Voltage‐Dependent Thresholds for Inducing Long‐Term Depression and Long‐Term Potentiation in Slices of Rat Visual Cortex.” Nature 347, no. 6288: 69–72. 1975639 10.1038/347069a0

[hipo70068-bib-0010] Bartesaghi, R. , and L. Ravasi . 1999. “Pyramidal Neuron Types in Field CA2 of the Guinea Pig.” Brain Research Bulletin 50, no. 4: 263–273. 10582524 10.1016/s0361-9230(99)00198-7

[hipo70068-bib-0011] Bear, M. F. , L. N. Cooper , and F. F. Ebner . 1987. “A Physiological Basis for a Theory of Synapse Modification.” Science 237, no. 4810: 42–48. 3037696 10.1126/science.3037696

[hipo70068-bib-0012] Berger, S. , D. P. Wolfer , O. Selbach , et al. 2006. “Loss of the Limbic Mineralocorticoid Receptor Impairs Behavioral Plasticity.” Proceedings of the National Academy of Sciences of the United States of America 103, no. 1: 195–200. 16368758 10.1073/pnas.0503878102 PMC1324975

[hipo70068-bib-0013] Bienenstock, E. L. , L. N. Cooper , and P. W. Munro . 1982. “Theory for the Development of Neuron Selectivity: Orientation Specificity and Binocular Interaction in Visual Cortex.” Journal of Neuroscience 2, no. 1: 32–48. 7054394 10.1523/JNEUROSCI.02-01-00032.1982 PMC6564292

[hipo70068-bib-0014] Boulanger, L. M. , P. J. Lombroso , A. Raghunathan , M. J. During , P. Wahle , and J. R. Naegele . 1995. “Cellular and Molecular Characterization of a Brain‐Enriched Protein Tyrosine Phosphatase.” Journal of Neuroscience 15, no. 2: 1532–1544. 7869116 10.1523/JNEUROSCI.15-02-01532.1995 PMC6577844

[hipo70068-bib-0015] Bowen, W. D. , B. N. Kirschner , A. H. Newman , and K. C. Rice . 1988. “Sigma Receptors Negatively Modulate Agonist‐Stimulated Phosphoinositide Metabolism in Rat Brain.” European Journal of Pharmacology 149, no. 3: 399–400. 2842167 10.1016/0014-2999(88)90678-4

[hipo70068-bib-0016] Burstein, S. R. , T. J. Williams , D. A. Lane , et al. 2013. “The Infulence of Reproductive Status and Acute Stress on the Levels of Phosphorylated Delta Opioid Receptor Immunoreactivity in Rat Hippocampus.” Brain Research 1518: 71–81. 23583481 10.1016/j.brainres.2013.03.051 PMC3764923

[hipo70068-bib-0017] Calfa, G. , W. Li , J. M. Rutherford , and L. Pozzo‐Miller . 2015. “Excitation/Inhibition Imbalance and Impaired Synaptic Inhibition in Hippocampal Area CA3 of Mecp2 Knockout Mice.” Hippocampus 25, no. 2: 159–168. 25209930 10.1002/hipo.22360 PMC4300269

[hipo70068-bib-0018] Carstens, K. E. , and S. M. Dudek . 2019. “Regulation of Synaptic Plasticity in Hippocampal Area CA2.” Current Opinion in Neurobiology 54: 194–199. 30120016 10.1016/j.conb.2018.07.008 PMC6361679

[hipo70068-bib-0019] Carstens, K. E. , B. R. Gloss , G. M. Alexander , and S. M. Dudek . 2021. “Modified Adeno‐Associated Virus Targets the Bacterial Enzyme Chondroitinase ABC to Select Mouse Neuronal Populations In Vivo Using the Cre‐LoxP System.” European Journal of Neuroscience 53, no. 12: 4005–4015. 33220084 10.1111/ejn.15050 PMC8137719

[hipo70068-bib-0020] Carstens, K. E. , D. J. Lustberg , E. K. Shaughnessy , K. E. McCann , G. M. Alexander , and S. M. Dudek . 2021. “Perineuronal Net Degradation Rescues CA2 Plasticity in a Mouse Model of Rett Syndrome.” Journal of Clinical Investigation 131, no. 16: e137221. 34228646 10.1172/JCI137221 PMC8363283

[hipo70068-bib-0021] Carstens, K. E. , M. L. Phillips , L. Pozzo‐Miller , R. J. Weinberg , and S. M. Dudek . 2016. “Perineuronal Nets Suppress Plasticity of Excitatory Synapses on CA2 Pyramidal Neurons.” Journal of Neuroscience 36, no. 23: 6312–6320. 27277807 10.1523/JNEUROSCI.0245-16.2016 PMC4899529

[hipo70068-bib-0022] Caruana, D. A. , and S. M. Dudek . 2020. “Adenosine A(1) Receptor‐Mediated Synaptic Depression in the Developing Hippocampal Area CA2.” Frontiers in Synaptic Neuroscience 12: 21. 32612520 10.3389/fnsyn.2020.00021 PMC7307308

[hipo70068-bib-0023] Cembrowski, M. S. , L. Wang , K. Sugino , B. C. Shields , and N. Spruston . 2016. “Hipposeq: A Comprehensive RNA‐Seq Database of Gene Expression in Hippocampal Principal Neurons.” eLife 5: e14997. 27113915 10.7554/eLife.14997 PMC4846374

[hipo70068-bib-0024] Chan, K. Y. , M. J. Jang , B. B. Yoo , et al. 2017. “Engineered AAVs for Efficient Noninvasive Gene Delivery to the Central and Peripheral Nervous Systems.” Nature Neuroscience 20, no. 8: 1172–1179. 28671695 10.1038/nn.4593 PMC5529245

[hipo70068-bib-0025] Chevaleyre, V. , and S. A. Siegelbaum . 2010. “Strong CA2 Pyramidal Neuron Synapses Define a Powerful Disynaptic Cortico‐Hippocampal Loop.” Neuron 66, no. 4: 560–572. 20510860 10.1016/j.neuron.2010.04.013 PMC2905041

[hipo70068-bib-0026] Collingridge, G. L. 2025. “Glutamate Receptors and Synaptic Plasticity in Health and Disease: A Personal Journey.” Hippocampus. 10.1002/hipo.70062 PMC12812506 41549054

[hipo70068-bib-0027] Collingridge, G. L. , S. J. Kehl , and H. McLennan . 1983. “Excitatory Amino Acids in Synaptic Transmission in the Schaffer Collateral‐Commissural Pathway of the Rat Hippocampus.” Journal of Physiology 334: 33–46. 6306230 10.1113/jphysiol.1983.sp014478 PMC1197298

[hipo70068-bib-0028] Cope, E. C. , A. D. Zych , N. J. Katchur , et al. 2022. “Atypical Perineuronal Nets in the CA2 Region Interfere With Social Memory in a Mouse Model of Social Dysfunction.” Molecular Psychiatry 27, no. 8: 3520–3531. 34183768 10.1038/s41380-021-01174-2 PMC8712624

[hipo70068-bib-0029] Crozier, R. A. , Y. Wang , C. H. Liu , and M. F. Bear . 2007. “Deprivation‐Induced Synaptic Depression by Distinct Mechanisms in Different Layers of Mouse Visual Cortex.” Proceedings of the National Academy of Sciences of the United States of America 104, no. 4: 1383–1388. 17227847 10.1073/pnas.0609596104 PMC1783104

[hipo70068-bib-0030] Cukier, H. N. , A. J. Griswold , N. K. Hofmann , et al. 2020. “Three Brothers With Autism Carry a Stop‐Gain Mutation in the HPA‐Axis Gene NR3C2.” Autism Research 13, no. 4: 523–531. 32064789 10.1002/aur.2269

[hipo70068-bib-0031] Dasgupta, A. , N. Baby , K. Krishna , et al. 2017. “Substance P Induces Plasticity and Synaptic Tagging/Capture in Rat Hippocampal Area CA2.” Proceedings of the National Academy of Sciences of the United States of America 114, no. 41: E8741–E8749. 28973908 10.1073/pnas.1711267114 PMC5642719

[hipo70068-bib-0032] Diethorn, E. J. , and E. Gould . 2023. “Development of the Hippocampal CA2 Region and the Emergence of Social Recognition.” Developmental Neurobiology 83, no. 5–6: 143–156. 37326250 10.1002/dneu.22919 PMC10529477

[hipo70068-bib-0033] Donegan, M. L. , F. Stefanini , T. Meira , J. A. Gordon , S. Fusi , and S. A. Siegelbaum . 2020. “Coding of Social Novelty in the Hippocampal CA2 Region and Its Disruption and Rescue in a 22q11.2 Microdeletion Mouse Model.” Nature Neuroscience 23, no. 11: 1365–1375. 33077947 10.1038/s41593-020-00720-5 PMC8861630

[hipo70068-bib-0034] Dudek, S. M. , G. M. Alexander , and S. Farris . 2016. “Rediscovering Area CA2: Unique Properties and Functions.” Nature Reviews Neuroscience 17, no. 2: 89–102. 26806628 10.1038/nrn.2015.22 PMC4856153

[hipo70068-bib-0035] Dudek, S. M. , G. M. Alexander , and S. Farris . 2023. “Introduction to the Special Issue on: A New View of Hippocampal Area CA2.” Hippocampus 33, no. 3: 127–132. 36826426 10.1002/hipo.23514

[hipo70068-bib-0036] Dudek, S. M. , and M. F. Bear . 1989. “A Biochemical Correlate of the Critical Period for Synaptic Modification in the Visual Cortex.” Science 246, no. 4930: 673–675. 2573152 10.1126/science.2573152

[hipo70068-bib-0037] Dudek, S. M. , and M. F. Bear . 1992. “Homosynaptic Long‐Term Depression in Area CA1 of Hippocampus and Effects of N‐Methyl‐D‐Aspartate Receptor Blockade.” Proceedings of the National Academy of Sciences of the United States of America 89, no. 10: 4363–4367. 1350090 10.1073/pnas.89.10.4363 PMC49082

[hipo70068-bib-0038] Dudek, S. M. , and M. F. Bear . 1993. “Bidirectional Long‐Term Modification of Synaptic Effectiveness in the Adult and Immature Hippocampus.” Journal of Neuroscience 13, no. 7: 2910–2918. 8331379 10.1523/JNEUROSCI.13-07-02910.1993 PMC6576673

[hipo70068-bib-0039] Dudek, S. M. , W. D. Bowen , and M. F. Bear . 1989. “Postnatal Changes in Glutamate Stimulated Phosphoinositide Turnover in Rat Neocortical Synaptoneurosomes.” Brain Research. Developmental Brain Research 47, no. 1: 123–128. 2567641 10.1016/0165-3806(89)90114-4

[hipo70068-bib-0040] Dudek, S. M. , and M. J. Friedlander . 1996. “Developmental Down‐Regulation of LTD in Cortical Layer IV and Its Independence of Modulation by Inhibition.” Neuron 16, no. 6: 1097–1106. 8663986 10.1016/s0896-6273(00)80136-1

[hipo70068-bib-0041] Eaton, S. A. , D. E. Jane , P. L. Jones , et al. 1993. “Competitive Antagonism at Metabotropic Glutamate Receptors by (S)‐4‐Carboxyphenylglycine and (RS)‐Alpha‐Methyl‐4‐Carboxyphenylglycine.” European Journal of Pharmacology 244, no. 2: 195–197. 8381746 10.1016/0922-4106(93)90028-8

[hipo70068-bib-0042] Farris, S. , Y. Wang , J. M. Ward , and S. M. Dudek . 2017. “Optimized Method for Robust Transcriptome Profiling of Minute Tissues Using Laser Capture Microdissection and Low‐Input RNA‐Seq.” Frontiers in Molecular Neuroscience 10: 185. 28659759 10.3389/fnmol.2017.00185 PMC5468370

[hipo70068-bib-0043] Farris, S. , J. M. Ward , K. E. Carstens , M. Samadi , Y. Wang , and S. M. Dudek . 2019. “Hippocampal Subregions Express Distinct Dendritic Transcriptomes That Reveal Differences in Mitochondrial Function in CA2.” Cell Reports 29, no. 2: 522–539.e526. 31597108 10.1016/j.celrep.2019.08.093 PMC6894405

[hipo70068-bib-0044] Franceschini, A. , M. Jin , C. W. Chen , L. Silvestri , A. Mastrodonato , and C. A. Denny . 2025. “Brain‐Wide Immunolabeling and Tissue Clearing Applications for Engram Research.” Neurobiology of Learning and Memory 218: 108032. 39922482 10.1016/j.nlm.2025.108032 PMC13202242

[hipo70068-bib-0045] Fregnac, Y. , J. P. Burke , D. Smith , and M. J. Friedlander . 1994. “Temporal Covariance of Pre‐ and Postsynaptic Activity Regulates Functional Connectivity in the Visual Cortex.” Journal of Neurophysiology 71, no. 4: 1403–1421. 8035224 10.1152/jn.1994.71.4.1403

[hipo70068-bib-0046] Gordon, J. A. , and M. P. Stryker . 1996. “Experience‐Dependent Plasticity of Binocular Responses in the Primary Visual Cortex of the Mouse.” Journal of Neuroscience 16, no. 10: 3274–3286. 8627365 10.1523/JNEUROSCI.16-10-03274.1996 PMC6579137

[hipo70068-bib-0047] Harris, E. P. , B. Kandemir , S. M. Jones , et al. 2025. “Mineralocorticoid Receptor Knockout Alters Hippocampal CA2 Neurons to Become Like Those in CA1.” Communications Biology 8, no. 1: 1037. 40640339 10.1038/s42003-025-08378-0 PMC12246475

[hipo70068-bib-0048] Harris, K. M. , and T. J. Teyler . 1984. “Developmental Onset of Long‐Term Potentiation in Area CA1 of the Rat Hippocampus.” Journal of Physiology 346: 27–48. 6699775 10.1113/jphysiol.1984.sp015005 PMC1199482

[hipo70068-bib-0049] Herman, J. P. , P. D. Patel , H. Akil , and S. J. Watson . 1989. “Localization and Regulation of Glucocorticoid and Mineralocorticoid Receptor Messenger RNAs in the Hippocampal Formation of the Rat.” Molecular Endocrinology 3, no. 11: 1886–1894. 2558306 10.1210/mend-3-11-1886

[hipo70068-bib-0050] Hitti, F. L. , and S. A. Siegelbaum . 2014. “The Hippocampal CA2 Region Is Essential for Social Memory.” Nature 508, no. 7494: 88–92. 24572357 10.1038/nature13028 PMC4000264

[hipo70068-bib-0051] Huber, K. M. , J. C. Roder , and M. F. Bear . 2001. “Chemical Induction of mGluR5‐ and Protein Synthesis—Dependent Long‐Term Depression in Hippocampal Area CA1.” Journal of Neurophysiology 86, no. 1: 321–325. 11431513 10.1152/jn.2001.86.1.321

[hipo70068-bib-0052] Huh, G. S. , L. M. Boulanger , H. Du , P. A. Riquelme , T. M. Brotz , and C. J. Shatz . 2000. “Functional Requirement for Class I MHC in CNS Development and Plasticity.” Science 290, no. 5499: 2155–2159. 11118151 10.1126/science.290.5499.2155 PMC2175035

[hipo70068-bib-0053] Hwang, P. M. , D. S. Bredt , and S. H. Snyder . 1990. “Autoradiographic Imaging of Phosphoinositide Turnover in the Brain.” Science 249, no. 4970: 802–804. 1975122 10.1126/science.1975122

[hipo70068-bib-0054] Ikeda, M. 1993. “Reduction of Phosphoinositide Hydrolysis by L‐Amino‐3‐Phosphonopropionate May Be Caused by the Inhibition of Synthesis of Phosphatidylinositols.” Neuroscience Letters 157, no. 1: 87–90. 8233038 10.1016/0304-3940(93)90649-6

[hipo70068-bib-0055] Ito, M. , and M. Kano . 1982. “Long‐Lasting Depression of Parallel Fiber‐Purkinje Cell Transmission Induced by Conjunctive Stimulation of Parallel Fibers and Climbing Fibers in the Cerebellar Cortex.” Neuroscience Letters 33, no. 3: 253–258. 6298664 10.1016/0304-3940(82)90380-9

[hipo70068-bib-0056] Kalman, B. A. , and R. L. Spencer . 2002. “Rapid Corticosteroid‐Dependent Regulation of Mineralocorticoid Receptor Protein Expression in Rat Brain.” Endocrinology 143, no. 11: 4184–4195. 12399411 10.1210/en.2002-220375

[hipo70068-bib-0057] Kanjhan, R. , A. M. Anselme , P. G. Noakes , and M. C. Bellingham . 2004. “Postnatal Changes in TASK‐1 and TREK‐1 Expression in Rat Brain Stem and Cerebellum.” Neuroreport 15, no. 8: 1321–1324. 15167558 10.1097/01.wnr.0000127462.15985.dc

[hipo70068-bib-0058] Kirkwood, A. , S. M. Dudek , J. T. Gold , C. D. Aizenman , and M. F. Bear . 1993. “Common Forms of Synaptic Plasticity in the Hippocampus and Neocortex In Vitro.” Science 260, no. 5113: 1518–1521. 8502997 10.1126/science.8502997

[hipo70068-bib-0059] Kohara, K. , M. Pignatelli , A. J. Rivest , et al. 2014. “Cell Type‐Specific Genetic and Optogenetic Tools Reveal Hippocampal CA2 Circuits.” Nature Neuroscience 17, no. 2: 269–279. 24336151 10.1038/nn.3614 PMC4004172

[hipo70068-bib-0060] Kwak, S. P. , P. D. Patel , R. C. Thompson , H. Akil , and S. J. Watson . 1993. “5'‐Heterogeneity of the Mineralocorticoid Receptor Messenger Ribonucleic Acid: Differential Expression and Regulation of Splice Variants Within the Rat Hippocampus.” Endocrinology 133, no. 5: 2344–2350. 8404687 10.1210/endo.133.5.8404687

[hipo70068-bib-0061] Laham, B. J. , E. J. Diethorn , and E. Gould . 2021. “Newborn Mice Form Lasting CA2‐Dependent Memories of Their Mothers.” Cell Reports 34, no. 4: 108668. 33503421 10.1016/j.celrep.2020.108668 PMC7985754

[hipo70068-bib-0062] Lee, H. K. , M. Barbarosie , K. Kameyama , M. F. Bear , and R. L. Huganir . 2000. “Regulation of Distinct AMPA Receptor Phosphorylation Sites During Bidirectional Synaptic Plasticity.” Nature 405, no. 6789: 955–959. 10879537 10.1038/35016089

[hipo70068-bib-0063] Lee, H. K. , K. Kameyama , R. L. Huganir , and M. F. Bear . 1998. “NMDA Induces Long‐Term Synaptic Depression and Dephosphorylation of the GluR1 Subunit of AMPA Receptors in Hippocampus.” Neuron 21, no. 5: 1151–1162. 9856470 10.1016/s0896-6273(00)80632-7

[hipo70068-bib-0064] Lee, S. E. , S. B. Simons , S. A. Heldt , et al. 2010. “RGS14 Is a Natural Suppressor of Both Synaptic Plasticity in CA2 Neurons and Hippocampal‐Based Learning and Memory.” Proceedings of the National Academy of Sciences of the United States of America 107, no. 39: 16994–16998. 20837545 10.1073/pnas.1005362107 PMC2947872

[hipo70068-bib-0065] Lein, E. S. , X. Zhao , and F. H. Gage . 2004. “Defining a Molecular Atlas of the Hippocampus Using DNA Microarrays and High‐Throughput In Situ Hybridization.” Journal of Neuroscience 24, no. 15: 3879–3889. 15084669 10.1523/JNEUROSCI.4710-03.2004 PMC6729356

[hipo70068-bib-0066] Leroy, F. , J. Park , A. Asok , et al. 2018. “A Circuit From Hippocampal CA2 to Lateral Septum Disinhibits Social Aggression.” Nature 564, no. 7735: 213–218. 30518859 10.1038/s41586-018-0772-0 PMC6364572

[hipo70068-bib-0067] Lorente de Nó, R. 1934. “Studies on the Structure of the Cerebral Cortex II. Continuation of the Study of the Ammonic System.” Journal für Psychologie und Neurologie 46: 113–177.

[hipo70068-bib-0068] Lyckman, A. W. , S. Horng , C. A. Leamey , et al. 2008. “Gene Expression Patterns in Visual Cortex During the Critical Period: Synaptic Stabilization and Reversal by Visual Deprivation.” Proceedings of the National Academy of Sciences 105, no. 27: 9409–9414. 10.1073/pnas.0710172105 PMC2453704 18606990

[hipo70068-bib-0069] Lynch, G. , J. Larson , S. Kelso , G. Barrionuevo , and F. Schottler . 1983. “Intracellular Injections of EGTA Block Induction of Hippocampal Long‐Term Potentiation.” Nature 305, no. 5936: 719–721. 6415483 10.1038/305719a0

[hipo70068-bib-0070] Mankin, E. A. , G. W. Diehl , F. T. Sparks , S. Leutgeb , and J. K. Leutgeb . 2015. “Hippocampal CA2 Activity Patterns Change Over Time to a Larger Extent Than Between Spatial Contexts.” Neuron 85, no. 1: 190–201. 25569350 10.1016/j.neuron.2014.12.001 PMC4392894

[hipo70068-bib-0071] Mattioni, L. , A. Barbieri , A. Grigoli , L. Balasco , Y. Bozzi , and G. Provenzano . 2024. “Alterations of Perineuronal Net Expression and Abnormal Social Behavior and Whisker‐Dependent Texture Discrimination in Mice Lacking the Autism Candidate Gene Engrailed 2.” Neuroscience 546: 63–74. 38537894 10.1016/j.neuroscience.2024.03.023

[hipo70068-bib-0072] McCann, K. E. , D. J. Lustberg , E. K. Shaughnessy , et al. 2021. “Novel Role for Mineralocorticoid Receptors in Control of a Neuronal Phenotype.” Molecular Psychiatry 26, no. 1: 350–364. 31745235 10.1038/s41380-019-0598-7 PMC7234915

[hipo70068-bib-0073] Mercer, A. , H. L. Trigg , and A. M. Thomson . 2007. “Characterization of Neurons in the CA2 Subfield of the Adult Rat Hippocampus.” Journal of Neuroscience 27, no. 27: 7329–7338. 17611285 10.1523/JNEUROSCI.1829-07.2007 PMC6794598

[hipo70068-bib-0074] Mower, G. D. , C. J. Caplan , W. G. Christen , and F. H. Duffy . 1985. “Dark Rearing Prolongs Physiological but Not Anatomical Plasticity of the Cat Visual Cortex.” Journal of Comparative Neurology 235, no. 4: 448–466. 3998219 10.1002/cne.902350404

[hipo70068-bib-0075] Mulkey, R. M. , and R. C. Malenka . 1992. “Mechanisms Underlying Induction of Homosynaptic Long‐Term Depression in Area CA1 of the Hippocampus.” Neuron 9, no. 5: 967–975. 1419003 10.1016/0896-6273(92)90248-c

[hipo70068-bib-0076] Nicoletti, F. , M. J. Iadarola , J. T. Wroblewski , and E. Costa . 1986. “Excitatory Amino Acid Recognition Sites Coupled With Inositol Phospholipid Metabolism: Developmental Changes and Interaction With Alpha 1‐Adrenoceptors.” Proceedings of the National Academy of Sciences of the United States of America 83, no. 6: 1931–1935. 2869493 10.1073/pnas.83.6.1931 PMC323198

[hipo70068-bib-0077] Nicoll, R. A. 2017. “A Brief History of Long‐Term Potentiation.” Neuron 93, no. 2: 281–290. 28103477 10.1016/j.neuron.2016.12.015

[hipo70068-bib-0078] Nicoll, R. A. 2025. “LTP: A Personal Journey and Beyond.” Hippocampus. 10.1002/hipo.70055 41479033

[hipo70068-bib-0079] Ochiishi, T. , Y. Saitoh , A. Yukawa , et al. 1999. “High Level of Adenosine A1 Receptor‐Like Immunoreactivity in the CA2/CA3a Region of the Adult Rat Hippocampus.” Neuroscience 93, no. 3: 955–967. 10473260 10.1016/s0306-4522(99)00179-7

[hipo70068-bib-0080] Otmakhov, N. , L. Khibnik , N. Otmakhova , et al. 2004. “Forskolin‐Induced LTP in the CA1 Hippocampal Region Is NMDA Receptor Dependent.” Journal of Neurophysiology 91, no. 5: 1955–1962. 14702333 10.1152/jn.00941.2003

[hipo70068-bib-0081] Pagani, J. H. , M. Zhao , Z. Cui , et al. 2015. “Role of the Vasopressin 1b Receptor in Rodent Aggressive Behavior and Synaptic Plasticity in Hippocampal Area CA2.” Molecular Psychiatry 20, no. 4: 490–499. 24863146 10.1038/mp.2014.47 PMC4562468

[hipo70068-bib-0082] Pannoni, K. E. , Q. S. Fischer , R. Tarannum , et al. 2025. “MCU Expression in Hippocampal CA2 Neurons Modulates Dendritic Mitochondrial Morphology and Synaptic Plasticity.” Scientific Reports 15, no. 1: 4540. 39915602 10.1038/s41598-025-85958-4 PMC11802895

[hipo70068-bib-0083] Pannoni, K. E. , D. Gil , M. L. Cawley , M. M. Alsalman , L. A. Campbell , and S. Farris . 2023. “Layer‐Specific Mitochondrial Diversity Across Hippocampal CA2 Dendrites.” Hippocampus 33, no. 3: 182–196. 36762797 10.1002/hipo.23512 PMC9974919

[hipo70068-bib-0084] Paul, S. , A. C. Nairn , P. Wang , and P. J. Lombroso . 2003. “NMDA‐Mediated Activation of the Tyrosine Phosphatase STEP Regulates the Duration of ERK Signaling.” Nature Neuroscience 6, no. 1: 34–42. 12483215 10.1038/nn989

[hipo70068-bib-0085] Paulsen, O. , Y. G. Li , O. Hvalby , P. Anderson , and T. V. Bliss . 1993. “Failure to Induce Long‐Term Depression by an Anti‐Correlation Procedure in Area CA1 of the Rat Hippocampal Slice.” European Journal of Neuroscience 5, no. 10: 1241–1246. 8275226 10.1111/j.1460-9568.1993.tb00909.x

[hipo70068-bib-0086] Pelkey, K. A. , R. Askalan , S. Paul , et al. 2002. “Tyrosine Phosphatase STEP Is a Tonic Brake on Induction of Long‐Term Potentiation.” Neuron 34, no. 1: 127–138. 11931747 10.1016/s0896-6273(02)00633-5

[hipo70068-bib-0087] Pizzorusso, T. , P. Medini , N. Berardi , S. Chierzi , J. W. Fawcett , and L. Maffei . 2002. “Reactivation of Ocular Dominance Plasticity in the Adult Visual Cortex.” Science 298, no. 5596: 1248–1251. 12424383 10.1126/science.1072699

[hipo70068-bib-0088] Ruzzo, E. K. , L. Perez‐Cano , J. Y. Jung , et al. 2019. “Inherited and De Novo Genetic Risk for Autism Impacts Shared Networks.” Cell 178, no. 4: 850–866.e826. 31398340 10.1016/j.cell.2019.07.015 PMC7102900

[hipo70068-bib-0089] Samadi, M. , C. A. Hales , D. J. Lustberg , et al. 2023. “Mechanisms of mGluR‐Dependent Plasticity in Hippocampal Area CA2.” Hippocampus 33, no. 6: 730–744. 36971428 10.1002/hipo.23529 PMC10213158

[hipo70068-bib-0090] Sejnowski, T. J. 1977a. “Statistical Constraints on Synaptic Plasticity.” Journal of Theoretical Biology 69, no. 2: 385–389. 592884 10.1016/0022-5193(77)90146-1

[hipo70068-bib-0091] Sejnowski, T. J. 1977b. “Storing Covariance With Nonlinearly Interacting Neurons.” Journal of Mathematical Biology 4, no. 4: 303–321. 925522 10.1007/BF00275079

[hipo70068-bib-0092] Seubert, P. , M. Baudry , S. Dudek , and G. Lynch . 1987. “Calmodulin Stimulates the Degradation of Brain Spectrin by Calpain.” Synapse 1, no. 1: 20–24. 2850618 10.1002/syn.890010105

[hipo70068-bib-0093] Simonnet, C. , M. Sinha , M. Goutierre , I. Moutkine , S. Daumas , and J. C. Poncer . 2023. “Silencing KCC2 in Mouse Dorsal Hippocampus Compromises Spatial and Contextual Memory.” Neuropsychopharm 48, no. 7: 1067–1077. 10.1038/s41386-022-01480-5 PMC10209115 36302847

[hipo70068-bib-0094] Simons, S. B. , D. A. Caruana , M. Zhao , and S. M. Dudek . 2011. “Caffeine‐Induced Synaptic Potentiation in Hippocampal CA2 Neurons.” Nature Neuroscience 15, no. 1: 23–25. 22101644 10.1038/nn.2962 PMC3245784

[hipo70068-bib-0095] Simons, S. B. , Y. Escobedo , R. Yasuda , and S. M. Dudek . 2009. “Regional Differences in Hippocampal Calcium Handling Provide a Cellular Mechanism for Limiting Plasticity.” Proceedings of the National Academy of Sciences of the United States of America 106, no. 33: 14080–14084. 19666491 10.1073/pnas.0904775106 PMC2729023

[hipo70068-bib-0096] Stanton, P. K. , and T. J. Sejnowski . 1989. “Associative Long‐Term Depression in the Hippocampus Induced by Hebbian Covariance.” Nature 339, no. 6221: 215–218. 2716848 10.1038/339215a0

[hipo70068-bib-0097] Talley, E. M. , G. Solorzano , Q. Lei , D. Kim , and D. A. Bayliss . 2001. “CNS Distribution of Members of the Two‐Pore‐Domain (KCNK) Potassium Channel Family.” Journal of Neuroscience 21, no. 19: 7491–7505. 11567039 10.1523/JNEUROSCI.21-19-07491.2001 PMC6762917

[hipo70068-bib-0098] Ter Horst, J. P. , M. van der Mark , J. Kentrop , et al. 2014. “Deletion of the Forebrain Mineralocorticoid Receptor Impairs Social Discrimination and Decision‐Making in Male, but Not in Female Mice.” Frontiers in Behavioral Neuroscience 8: 26. 24567706 10.3389/fnbeh.2014.00026 PMC3915243

[hipo70068-bib-0099] Thibault, O. , M. Joly , D. Muller , F. Schottler , S. Dudek , and G. Lynch . 1989. “Long‐Lasting Physiological Effects of Bath Applied N‐Methyl‐D‐Aspartate.” Brain Research 476, no. 1: 170–173. 2563332 10.1016/0006-8993(89)91553-9

[hipo70068-bib-0100] van Strien, N. M. , N. L. Cappaert , and M. P. Witter . 2009. “The Anatomy of Memory: An Interactive Overview of the Parahippocampal‐Hippocampal Network.” Nature Reviews. Neuroscience 10, no. 4: 272–282. 19300446 10.1038/nrn2614

[hipo70068-bib-0101] Wersinger, S. R. , E. I. Ginns , A. M. O'Carroll , S. J. Lolait , and W. S. Young . 2002. “Vasopressin V1b Receptor Knockout Reduces Aggressive Behavior in Male Mice.” Molecular Psychiatry 7, no. 9: 975–984. 12399951 10.1038/sj.mp.4001195

[hipo70068-bib-0102] Wyszynski, M. , V. Kharazia , R. Shanghvi , et al. 1998. “Differential Regional Expression and Ultrastructural Localization of Alpha‐Actinin‐2, a Putative NMDA Receptor‐Anchoring Protein, in Rat Brain.” Journal of Neuroscience 18, no. 4: 1383–1392. 9454847 10.1523/JNEUROSCI.18-04-01383.1998 PMC6792723

[hipo70068-bib-0103] Young, W. S. , J. Li , S. R. Wersinger , and M. Palkovits . 2006. “The Vasopressin 1b Receptor Is Prominent in the Hippocampal Area CA2 Where It Is Unaffected by Restraint Stress or Adrenalectomy.” Neuroscience 143, no. 4: 1031–1039. 17027167 10.1016/j.neuroscience.2006.08.040 PMC1748954

[hipo70068-bib-0104] Zhao, M. , Y. S. Choi , K. Obrietan , and S. M. Dudek . 2007. “Synaptic Plasticity (And the Lack Thereof) in Hippocampal CA2 Neurons.” Journal of Neuroscience 27, no. 44: 12025–12032. 17978044 10.1523/JNEUROSCI.4094-07.2007 PMC6673350

[hipo70068-bib-0105] Zhao, X. , E. S. Lein , A. He , S. C. Smith , C. Aston , and F. H. Gage . 2001. “Transcriptional Profiling Reveals Strict Boundaries Between Hippocampal Subregions.” Journal of Comparative Neurology 441, no. 3: 187–196. 11745644 10.1002/cne.1406

